# Vitamin D levels in patients with ankylosing spondylitis: Is it related to disease activity?

**DOI:** 10.12669/pjms.345.15739

**Published:** 2018

**Authors:** Burhan Fatih Kocyigit, Ahmet Akyol

**Affiliations:** 1 Dr. Burhan Fatih Kocyigit, Department of Physical Medicine and Rehabilitation, Kahramanmaras Sutcu Imam University School of Medicine, Kahramanmaras, Turkey; 2 Dr. Ahmet Akyol, Department of Physical Medicine and Rehabilitation, Nizip State Hospital; Gaziantep, Turkey

**Keywords:** Ankylosing Spondylitis, Vitamin D, Bone bineral density, Disease activity

## Abstract

**Objective:**

Ankylosing Spondylitis (AS) is an inflammatory rheumatic disease that mainly affects the axial spine. Osteopenia and osteoporosis are the main complications of AS. Vitamin D has functions on the immune system. In this study, we aimed to compare vitamin D levels and Bone Mineral Density (BMD) values between AS patients and controls.

**Methods:**

A total of 68 patients with axial AS and 34 healthy controls were enrolled in this study conducted between March 2018 and May 2018. Vitamin D concentrations, BMD values, disease activity, back mobility, functionality and radiologic damage were evaluated.

**Results:**

Vitamin D concentrations, the total BMD-femur and BMD-femur neck values were significantly lower in AS patients (p = 0.001, p = 0.011 and p = 0.003). No significant correlations were detected between vitamin D levels and BMD-femur total, BMD-femur neck values, disease activity, back mobility, functionality and radiologic damage scores (p > 0.05). Disease activity parameters were significantly and negatively correlated with total BMD-femur and BMD-femur neck values (p < 0.05).

**Conclusion:**

Our study demonstrates that AS patients have lower vitamin D levels, total BMD-femur and BMD-femur neck values. Higher disease activity increases bone loss in AS. Regular measurement of BMD and vitamin D should be kept in mind when planning a treatment in AS.

## INTRODUCTION

Spondyloarthropathies (SpA) are a group of diseases in which rheumatoid factor and anti-nuclear antibodies are negative and presenting with spinal, articular, and extra-articular symptoms.^1^ Ankylosing Spondylitis (AS) is a major rheumatic disorder of the (SpA) group that mainly influences the axial spine and sacroiliac joints. New bone formation, syndesmophytes, and ankylosis of the spine lead to pain, spinal deformity, fracture, and disability.^1^ Inflammatory bowel disease, lung abnormalities, uveitis, cardiac abnormalities, psoriasis and amyloidosis are extra-articular manifestations of AS.^2^ Osteopenia and osteoporosis, which are the main complications of AS substantially increase the risk of spinal fractures.^3^,^4^ Inflammatory pathways play a role in the complex pathophysiologic mechanism of osteopenia and osteoporosis in AS.^5^-^7^ Additionally, immobilization, drugs, genetic variations and hormonal alterations are potential factors that influence bone metabolism.^8^,^9^

Vitamin D, which is defined as a hormone, has functions on the calcium homeostasis and bone metabolism, as well as on the immune system.^10^ The inhibitor and activator effects of vitamin D on the immune system have been demonstrated.^11^ Vitamin D influences the innate and adaptive immune system cells, which contribute its immuno-regulatory role.^12^ Receptors of vitamin D are expressed on the macrophages, lymphocytes, and dendritic cells. Vitamin D decreases levels of proinflammatory cytokines and inhibits the immune activity of macrophages.^10^ Vitamin D has been associated with various diseases such as cardiovascular disorders, infections, malignancy, diabetes mellitus, inflammatory disorders, fibromyalgia, and multiple sclerosis.^13^-^15^ Conflicting results have been demonstrated regarding the link between vitamin D levels and AS.^16^

Our primary aim was to compare vitamin D levels and Bone Mineral Density (BMD) values between AS and control groups. The secondary aim was to evaluate the associations between vitamin D and disease activity, functionality, and radiologic damage in AS.

## METHODS

This case control study was conducted between March 2018 and May 2018. A total of 76 patients with axial AS who presented to physical medicine and rehabilitation (outpatient) clinic and 34 healthy controls were evaluated. The patients with AS who met the modified New York criteria were included the study.^17^

Exclusion criteria were a history of malignancy, metabolic bone diseases, recent fractures, malnutrition, chronic infection, inflammatory bowel disease, diabetes mellitus, thyroid dysfunction, cardiac and renal disease, pregnancy, drug use affecting metabolism of bone such as bisphosphonates, vitamin D supplementations and glucocorticoids, and breast-feeding.

Considering the exclusion criteria, a total of 68 AS patients were included and two groups were formed according to medication use, patients receiving tumor necrosis factor alpha (TNF-α) inhibitor and those receiving non-steroidal anti-inflammatory drug (NSAID) groups. *Patients in our study did not use DMARD (sulfasalazine, methotrexate) or secukinumab*.

### Data Sources &Measurement

Data were recorded including age, sex, disease duration, and body mass index (BMI). Disease activity was evaluated using the Bath Ankylosing Spondylitis Disease Activity Index (BASDAI) and Ankylosing Spondylitis Disease Activity Index-CRP (ASDAS-CRP).^18^,^19^ Back mobility was evaluated with Bath Ankylosing Spondylitis Metrology Index (BASMI), and functional ability was evaluated with Bath Ankylosing Spondylitis Functional Index (BASFI).^20^

### Radiologic Assessment

Radiologic changes were assessed by using the Modified Stoke Ankylosing Spondylitis Spinal Score (mSASSS).^21^,^22^ Radiographs were scored by a radiologist who was blinded to the patients’ identity. Dual energy X-ray absorptiometry (DEXA) (HOLOGIC 4500 A) was used to measure BMD in the lumbar spine (L1 - L4 anterior - posterior), total femur, and femur neck. The results are expressed as g/cm^2^.

### Radiologic assessments were performed at the same day of the physical examination

### Laboratory Assessment

On the same day of physical examination, blood samples were obtained from all participants between 8.00 – 9.00 AM after an over-night fast. C-reactive protein (CRP) (Cobas Integra 400 plus, Rotkreuz, Switzerland) and the erythrocyte sedimentation rate (ESR) (EventusVacuPlus ESR 100, Ankara, Turkey) were analyzed using standard laboratory techniques. Serum 25-hydroxyvitamin D (25’OH vit D) concentrations were measured using Enzyme-Linked Immunosorbent Assay (ELISA); and the results are expressed in units of ng/mL.

### Ethical Consideration

The Medical Ethics Committee of Kahranmaras Sutcu Imam University has approved this study (approval date: 31.01.2018; approval number: 14).

### Statistical Analysis

Statistical Package for Social Sciences for Windows version 20.0 package program (SPSS Inc., Chicago, IL, USA) was used to perform statistical analysis. Mean ± standard deviation, median (minimum-maximum), and number were used for the expression of results. Distribution of data was assessed using the Shapiro-Wilk test. The Chi-square test was performed to detect the differences in categorical variables between the two groups. A comparison of independent groups was performed using the independent sample t test or the Mann-Whitney U test according to the distribution of data. Spearman’s rho test was performed for the correlation analysis. The statistical significance level was considered as p < 0.05.

## RESULTS

In this study, 68 patients with axial AS and 34 healthy controls were enrolled. The mean ages in the patient and control groups were 41.51 ± 10.89 and 39.11 ± 6.57 years respectively. The mean BMI of the patient and control groups were 27.20 ± 4.38 kg/m^2^and 26.11 ± 3.81 kg/m^2^ respectively. No significant differences were detected between the patients and healthy controls in age, sex and BMI (p > 0.05) (Table-I). No significant differences were detected in the socio-demographic data between the TNF-α inhibitor (n=32) and NSAIDs (n=36) groups (p > 0.05).

**Table-I:** Demographic data of the patient and control groups.

	Patient Group (n = 68)	Control Group (n = 34)	P
Age	41.51± 10,89	39.11± 6.57	0.241
*Sex*			
Female	17	11	0.433
Male (n)	51	23	
BMI	27.20 ± 4.38	26.11± 3.81	0.220

***Abbreviations***: n: number; BMI: body mass index

The 25’OH vit D concentration, the total BMD-femur and BMD-femur neck values were significantly lower in the patient group (p = 0.001, p = 0.011 and p = 0.003). No significant difference was detected between the groups in BMD-lumbar spine values (p = 0.867) (Table-II).

**Table-II:** Comparison of bone mineral density and laboratory parameters between patient and control groups.

	Patient Group (n = 68) Median-min-max	Control Group (n = 34) Median-min-max	p
25’OH Vitamin D (ng / ml)	14.58-1.02 - 50	20.20-4.02-68	0.001
BMD – lumbar spine (g / cm^2^)	0.98-0.62-1.90	0.97-0.89-1.22	0.867
BMD – femur total (g / cm^2^)	0.91-0.65-1.24	0.98-0.83-1.27	0.011
BMD – femur neck (g / cm^2^)	0.83-0.55-1.74	0.91-0.80-1.30	0.003
ESR (mm / h)	9-1-47	5-1-35	0.001
CRP (mg / L)	5.88-0.09-45.30	1.19-0.03-11.40	< 0.001

***Abbreviations***: n: number; min: minimum; max: maximum; BMD: bone mineral density; ESR: erythrocyte sedimentation rate; CRP: C - reactive protein

No significant differences were detected between the TNF-α inhibitor and NSAID groups in terms of 25’OH vit D concentrations, BMD-lumbar spine, total BMD-femur, BMD-femur neck values, and ESR and CRP levels (p> 0.05).

No significant correlations were detected between BMD-lumbar spine values and ESR, CRP, BASDAI, and ASDAS in the patient group. On the other hand, CRP and ASDAS-CRP were significantly and negatively correlated with BMD-femur total values (r = -0.314, p = 0.009 and r = -0.292, p = 0.016). Additionally, ESR, CRP, and ASDAS-CRP were significantly and negatively correlated with BMD-femur neck values (r = -0.339, p = 0.005; r = -0.384, p = 0.001 and r = -0.303, p = 0.012).

25’OH vit D concentrations were significantly and negatively correlated with BMD-lumbar spine in the patient group (r = -0.258, p = 0.035). No significant correlations were detected between 25’OH vit D concentrations and symptom duration, BASDAI, ASDAS-CRP, BASMI, BASFI, mSASSS, VAS, ESR, CRP, total BMD-femur and BMD-femur neck in the patient group (p > 0.05) (Table-III).

**Table-III:** Correlations of clinic and laboratory parameters with 25-hydroxyvitamin D.

	rho	P
***Symptom duration***	0.130	0.294
BASDAI	-0.099	0.424
ASDAS	-0.102	0.411
BASMI	0.110	0.375
BASFI	0.017	0.888
MSASSS	0.053	0.669
ESR (mm/h)	-0.052	0.676
CRP (mg/L)	-0.074	0.550
BMD - lumbar spine (g/cm^2^)	-0.258	0.035
BMD - femurtotal (g/cm^2^)	-0.017	0.894
BMD - femur neck (g/cm^2^)	-0.200	0.105

**Abbreviations BASDAI:** Bath Ankylosing Spondylitis Disease Activity Index; **ASDAS:** Ankylosing Spondylitis Disease Activity Index; **BASFI:** Bath Ankylosing Spondylitis Functional Index; **BASMI:** Bath Ankylosing Spondylitis Metrology Index; **BASRI:** Bath Ankylosing Spondylitis Radiology Index; **MSASSS:** Modified Stoke Ankylosing Spondylitis Spinal Score; **VAS:** Visual Analog Scale; **ESR:** erythrocyte sedimentation rate; **CRP:** C - reactive protein; **BMD:** bone mineral density.

## DISCUSSION

Our study demonstrates that plasma concentrations of 25’OH vit D were significantly lower in patients with AS. On the other hand, no significant difference was detected between the TNF-α inhibitor and NSAID groups in terms of 25’OH vit D, and no significant correlations were detected between 25’OH vit D concentrations and disease activity, spine mobility, and radiologic changes in patients with AS. Vitamin D decreases the production of proinflammatory cytokines by inhibiting T helper-1 and T helper-17 cell activity. Additionally, it increases the anti-inflammatory response by activating T helper-2 and regulator T cell responses.^23^ After the discovery of the immunomodulatory functions of vitamin D, the role of vitamin D on the etiopathogenesis of rheumatic disorders has begun to attract interest. Consistent with our results, Mermerci et al.^9^, Erten et al.^24^, and Lange et al.^25^ reported decreased levels of vitamin D in AS. In contrast, Yazmalar et al.^10^ and Durmus et al.^26^ found no differences in vitamin D concentrations between AS and control groups. In the literature, the results of studies investigating the vitamin D and disease activity link are heterogeneous. Similar to our results, Yazmalar et al.^10^ detected no association between vitamin D concentrations and BASDAI scores. Mermerci et al.^9^ reported no significant correlation between vitamin D concentrations and ESR, CRP levels, and BASDAI scores. Arends et al.^27^ found no association between vitamin D and BASDAI, BASFI, BASMI scores, ESR, and CRP levels. However, in some studies significant inverse correlations have been detected between vitamin D levels and disease activity markers.^24^,^26^

There may be several explanations for the differences in the above-mentioned studies. In many studies, BASDAI, which is a self-reported and subjective scale, was used to assess disease activity. Widespread pain caused by vitamin D deficiency may lead to increased BASDAI scores. For this reason, we evaluated disease activity using ASDAS-CRP, which is a composite scale, as well as BASDAI. Sample sizes, ethnicity, vitamin D supplementation use, and seasonal variations may also affect the results. In many studies, heterogeneity has been observed in terms of drug use, duration of disease, and severity of disease.

In our study, total BMD-femur and BMD-femur neck values were significantly lower in the patient group. No significant difference was detected in the BMD-lumbar spine. Meirelles et al.^27^ and Jun et al.^28^ reported lower lumbar spine and proximal femur BMD scores in AS. Frank et al.^29^ found no significant difference in the lumbar spine BMD values, but total hip and femur neck BMD values were found to be decreased in patients with AS. Mermerci et al.^9^ reported lower BMD values in the femur neck and total femur in AS. Although they found a difference in lateral lumbar spine measurements, they reported no significant difference in anteroposterior measurements. DEXA is a simple method and widely used throughout the world for evaluating BMD. However, DEXA includes some limitations for evaluating BMD in patients with AS. In particular, lumbar spine DEXA measurements may incorrectly present increased BMD values due to new bone formation, syndesmophytes, facet joint fusion, and ligament ossification.^30^,^31^ AS is a disease characterized by new bone formation in the spinal column. DEXA may give false results in the lumbar region due to new bone formations. For this reason, femur neck BMD measurements instead of lumbar spine BMD measurements should be used for the diagnosis and follow-up of osteoporosis in AS patients. Osteoporosis is an important and frequent complication in patients with AS. Different factors such as immobilization due to pain, inflammation, cytokines, drugs affecting bone metabolism, and genetic factors may influence BMD in AS.^9^

In our study, clinical and laboratory disease activity indicators were correlated with BMD in patients with AS. CRP and ASDAS were significantly and negatively correlated with total BMD-femur and BMD-femur neck values. Additionally, the ESR was negatively correlated with BMD-femur neck values. In agreement with our results, it has been reported that bone loss increases when disease activity is high.^25^,^32^,^33^ In contrast, some researchers have suggested that there was no relationship between BMD values and disease activity.^34^,^35^ Proinflammatory cytokines are associated with bone loss, but evidence is still limited for this link. Inflammation may affect bone metabolism via proinflammatory cytokines which can increase osteoclastic activity.^36^ TNF-α, interleukin 1 (IL-1), and interleukin 6 (IL-6) concentrations have been found to associate with bone turnover markers such as pyridinoline, deoxypyridinoline, and osteocalcin in AS. Additionally, interleukin 17 (IL-17) which is an important cytokine in AS pathogenesis has been demonstrated to stimulate bone erosion by changing the receptor activator of nuclear factor-κB ligand and osteoprotegerin balance.^37^ Connections between the proinflammatory cytokines and remodeling of bone lead to bone loss in AS. Proinflammatory cytokines such as TNF-α and the transcription factor-like receptor activator of the nuclear factor kappa ligand stimulate the osteoclastogenesis which cause osteoporosis and fragility fractures.^38^ In this study, no significant differences were detected between the TNF-α inhibitor and NSAID groups in BMD values. These results may be due to the lack of differences in vitamin D, ESR, and CRP levels between the two groups.

### Limitations of the study

The sample size is small. The activity level, dietary habits and sunlight exposure frequencies of the patient and control groups were not evaluated. This study has a cross-sectional design and patients were not followed-up prospectively. *We did not evaluate calcium, phosphorus, alkaline phosphatase (ALP) and parathormone (PTH) levels in the study*. *HLA-B27 positivity was not assessed in the study*. Finally, bone turnover markers were not measured.

## CONCLUSION

This study suggests low vitamin D levels in AS and vitamin D was not associated with disease activity, spinal mobility, and radiologic damage. Total femur and femur neck bone loss was higher than the control group. Due to the spinal involvement, proximal femur BMD measurement is more convenient for diagnosis and follow-up of osteoporosis in AS patients. Higher disease activity increases bone loss and the reduction of BMD starts in the early phase of AS and continues throughout the disease. For this reason, regardless of age and sex, evaluation of bone loss is crucial in the follow-up of patients. Physicians should be aware of this fact when planning treatment. Regular measurement of BMD and vitamin D is important to prevent fractures that may cause vital complications and disability. Vitamin D is a potential factor in the pathogenesis of AS. Monitoring vitamin D levels may have benefits in controlling inflammation and disease activity.

### Authors Contribution

**BFK, AA:** Conceived, designed and did statistical analysis & editing of manuscript

**BFK, AA:** Did data collection and manuscript writing.

**BFK, AA:** Did review and final approval of manuscript.
